# A novel melting temperature mapping method may improve the prediction of postoperative intra-abdominal infection after pancreatoduodenectomy

**DOI:** 10.1093/bjs/znaf222

**Published:** 2025-10-24

**Authors:** Haruyoshi Tanaka, Mina Fukasawa, Nana Kimura, Kosuke Mori, Koshi Matsui, Ayaka Itoh, Katsuhisa Hirano, Toru Watanabe, Yoshihiro Shirai, Kentaro Nagaoka, Kazuto Shibuya, Isaku Yoshioka, Yoshihiro Yamamoto, Hideki Niimi, Tsutomu Fujii

**Affiliations:** Department of Surgery and Science, Faculty of Medicine, Academic Assembly, University of Toyama, Toyama, Japan; Department of Surgery, Nagoya University Hospital, Nagoya, Japan; Department of Surgery and Science, Faculty of Medicine, Academic Assembly, University of Toyama, Toyama, Japan; Department of Surgery and Science, Faculty of Medicine, Academic Assembly, University of Toyama, Toyama, Japan; Department of Surgery and Science, Faculty of Medicine, Academic Assembly, University of Toyama, Toyama, Japan; Department of Surgery and Science, Faculty of Medicine, Academic Assembly, University of Toyama, Toyama, Japan; Department of Surgery and Science, Faculty of Medicine, Academic Assembly, University of Toyama, Toyama, Japan; Department of Surgery and Science, Faculty of Medicine, Academic Assembly, University of Toyama, Toyama, Japan; Department of Surgery and Science, Faculty of Medicine, Academic Assembly, University of Toyama, Toyama, Japan; Department of Surgery and Science, Faculty of Medicine, Academic Assembly, University of Toyama, Toyama, Japan; Department of Clinical Infectious Diseases, Faculty of Medicine, Academic Assembly, University of Toyama, Toyama, Japan; Department of Surgery and Science, Faculty of Medicine, Academic Assembly, University of Toyama, Toyama, Japan; Department of Surgery and Science, Faculty of Medicine, Academic Assembly, University of Toyama, Toyama, Japan; Department of Clinical Infectious Diseases, Faculty of Medicine, Academic Assembly, University of Toyama, Toyama, Japan; Department of Clinical Laboratory and Molecular Pathology, Faculty of Medicine, Academic Assembly, University of Toyama, Toyama, Japan; Department of Surgery and Science, Faculty of Medicine, Academic Assembly, University of Toyama, Toyama, Japan

*Dear Editor,*

Postoperative intra-abdominal infection (POAI) remains a major challenge after pancreatoduodenectomy (PD)^1^. POAI after PD is predominantly caused by clinically relevant postoperative pancreatic fistula (CR-POPF), which is diagnosed based on the drainage fluid amylase (DFA) level^1^. Although appropriate drain management, including early removal of prophylactic drains, may reduce the incidence of POPF, delayed-onset POAI remains a significant concern^2,3^. Conventional tools for predicting POAI, such as Gram staining and bacterial culture, have limitations because Gram staining provides only broad bacterial classification and culture results require several days to become available^4,5^. To address these limitations, we developed a melting temperature (Tm) mapping method, a polymerase chain reaction (PCR)-based technique that enables rapid identification and quantification of causative bacteria within as little as 3 hours^6^. This study aimed to evaluate the potential clinical utility of Tm mapping in the management of POAI after PD.

After excluding one patient who underwent total pancreatectomy, 42 patients who underwent PD were analysed. In total, 81 drainage fluid samples collected around the pancreatico-jejunostomy on postoperative days (PODs) 1 and 3 were analysed using Tm mapping (see *Supplementary Methods*). The accuracy of Tm mapping was assessed using conventional bacterial culture as the reference standard. POAI was defined as either CR-POPF or intra-abdominal fluid collection or an abscess of Clavien–Dindo grade II or higher^1,7^.

Patient characteristics and postoperative outcomes are shown in *Table S1*. Among the 27 patients who developed POAI, four had intra-abdominal abscesses requiring image-guided drainage; no postoperative organ failure or 90-day mortality occurred. Based on receiver operating characteristic analysis, a Tm-mapping bacterial load of >1000 colony-forming units per millilitre (CFU/ml) on POD3 optimized the prediction of POAI (*Fig. S1*). When this threshold was used, Tm mapping demonstrated moderate diagnostic accuracy as a quantitative test (*Supplementary Results*). Causative bacterial species were also identified in six samples on POD1 and 12 on POD3, corresponding to 14 patients (*Fig. 1a*).

**Fig. 1:**
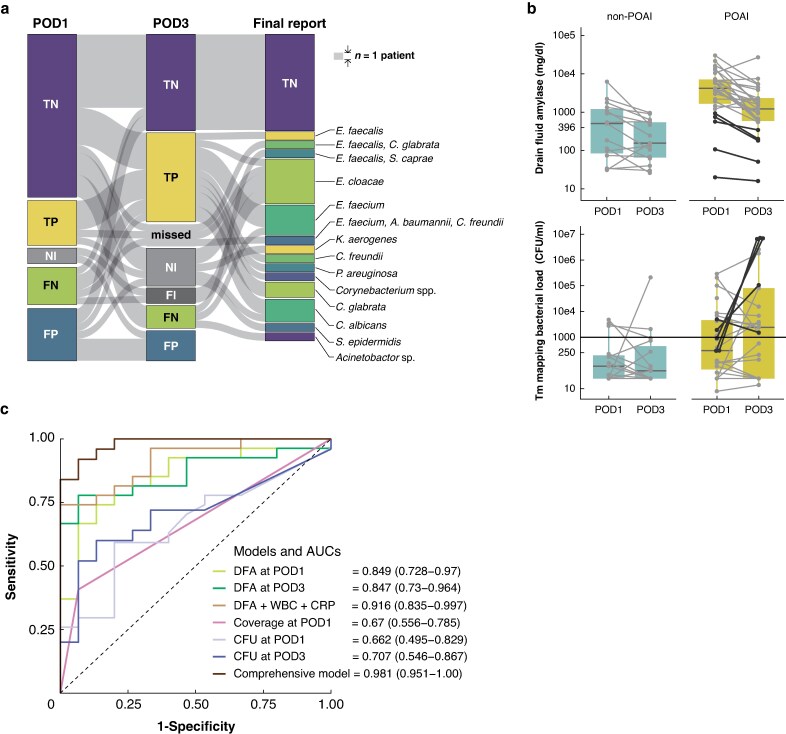
Performance of Tm mapping and its combination with clinical variables in predicting postoperative intra-abdominal infection (POAI) after pancreatoduodenectomy (PD) **a** Sankey plot illustrating the diagnostic accuracy of the Tm mapping method at postoperative days (PODs) 1 and 3, with bacterial culture results used as the reference standard. Each flow path represents the diagnostic performance of Tm mapping on POD1 and POD3, with the height proportional to the number of patients. The rightmost boxes represent culture outcomes. The height of the box at the upper right corresponds to that of a single patient (*n* = 1). TP indicates true positive; TN, true negative; FP, false positive; FN, false negative; NI, not identified by Tm mapping; FI, falsely identified (that is bacterial species detected by Tm mapping were different from those detected by culture test). Note that all tests in which the bacterial load was measured at ≤1000 CFU/ml by Tm mapping were categorized as negative. Each identified bacterial genus and species is listed in the *Supplementary Results*. **b** Boxplots of drain fluid amylase levels and bacterial loads quantified by Tm mapping on POD1 and POD3. The dots and connecting lines represent individual patient values. The black dots and lines indicate five patients who developed POAI without clinically relevant postoperative pancreatic fistula. **c** Receiver operating characteristic curves illustrating the predictive performance of generalized linear regression models for predicting POAI. Two models are multivariable models: the clinical model (camel line), which includes DFA, white blood cell count (WBC), and C-reactive protein (CRP) on POD3; and the comprehensive model (brown line), which incorporates those clinical variables plus bacterial load on POD3 and antibiotic coverage on POD1. AUC = area under the curve.

We next compared the predictive value of Tm mapping and DFA. Both the Tm-mapping bacterial load and the DFA were greater in the POAI group. Notably, bacterial loads generally increased from POD1 to POD3 only in the POAI group, whereas DFA decreased regardless of POAI status (*Fig. 1b*). In particular, five patients developed POAI without CR-POPF; all five had bacterial loads >1000 CFU/ml on POD3, suggesting that the bacterial load complements DFA for POAI prediction (black dots and lines in *Fig. 1b*).

Guided by the data in *Table S2*, in which several clinical parameters, ineffective antibiotic coverage, and the Tm-mapping bacterial load were identified as risk factors for POAI, we developed a novel model that incorporated these variables to predict POAI (*Supplementary Results*). The comprehensive model achieved an area under the curve of 0.981, exceeding those of the other models based solely on clinical variables from POD3 (*Fig. 1c*).

This study demonstrated two key potential advantages of the Tm mapping method in postoperative management. First, this method can support decisions regarding early drain removal. Even if DFA is low, retaining drains may be prudent when bacterial loads are >1000 CFU/ml on POD3 or when species intrinsically resistant to prophylactic antibiotics are identified. The observation that some patients with high bacterial loads developed POAI despite a decline in DFA from POD1 to POD3 underscores the value of rapid bacterial testing alongside clinical parameters. Second, Tm mapping can guide the prompt initiation of targeted antimicrobial therapy and help avoid unnecessary antibiotic use when the results are negative. A notable example is the rapid discrimination of closely related species with distinct resistance profiles. *Enterococcus faecalis* and *E. faecium* cannot be distinguished by Gram staining alone but differ in resistance patterns^8^. The diagnostic lag of approximately 1 week with culture also limits timely treatment decisions. Similarly, early identification of *Pseudomonas aeruginosa* among hundreds of species of Gram-negative rods will also support prompt antipseudomonal therapy and enhance precision in postoperative infection control^9^.

To our knowledge, this is the first study to evaluate a PCR-based bacterial identification method specifically in a surgical setting. Although several PCR-based platforms have been developed for bloodstream infections, their clinical use remains limited due to high costs, narrow detection spectra, and reliance on custom-designed fluorescent probes^10^. In contrast, our technology enables the identification and quantification of more than 100 bacterial species using only seven pairs of universal primers, offering broader coverage at a significantly lower cost. This simplicity and rapid turnaround make Tm mapping an attractive option for infection control in surgical patients.

The next step towards the clinical application of Tm mapping will be overcoming the limited ability to detect multiple species present in similar proportions. Rapid ancillary tests are needed in that case. Other limitations include the observational design and limited statistical power of the study. Interventional studies to validate the efficacy of postoperative management using ‘real-time’ Tm mapping are warranted.

## Supplementary Material

znaf222_Supplementary_Data

## Data Availability

The data used in the study are available from the first author upon reasonable request.
