# Supplementary material for: A novel melting temperature mapping method may improve the prediction of postoperative intra-abdominal infection after pancreatoduodenectomy
Source: Br J Surg. 2025 Oct 24;112(10):znaf222. doi: 10.1093/bjs/znaf222 (PMC12550889; doi:10.1093/bjs/znaf222)
Supplement: znaf222_Supplementary_Data [file znaf222_supplementary_data.docx]

**A novel melting temperature mapping method may improve the prediction of postoperative abdominal infection after pancreatoduodenectomy**

Haruyoshi Tanaka^*1,2^, Mina Fukasawa ^*1^, Nana Kimura^1^, Kosuke Mori^1^, Koshi Matsui^1^, Ayaka Itoh^1^, Katsuhisa Hirano^1^, Toru Watanabe^1^, Yoshihiro Shirai^1^, Kentaro Nagaoka^3^, Kazuto Shibuya^1^, Isaku Yoshioka^1^, Yoshihiro Yamamoto^3^, Hideki Niimi^4^, and Tsutomu Fujii^1^

^1^Department of Surgery and Science, Faculty of Medicine, Academic Assembly, University of Toyama, Toyama, Japan

^2^Department of Surgery, Nagoya University Hospital, Nagoya, Japan

^3^Department of Clinical Infectious Diseases, Faculty of Medicine, Academic Assembly, University of Toyama, Toyama, Japan

^4^Department of Clinical Laboratory and Molecular Pathology, Faculty of Medicine, Academic Assembly, University of Toyama, Toyama, Japan

^*^These authors contributed equally.

**Corresponding author:** Tsutomu Fujii, Department of Surgery and Science, Faculty of Medicine, Academic Assembly, University of Toyama, 2630 Sugitani, Toyama 930-0194, JAPAN

**ORCID ID:** 0000-0003-0625-1911

**Twitter:** not applicable

**Supplementary Materials - Index**

| **Supplementary Methods** |  |
| --- | --- |
| Patient Enrolment and Data Collection | *page 3* |
| Sample Collection and Handling | *page 3* |
| Melting Temperature (Tm) Mapping Method | *page 4* |
| Statistical Analysis | *page 4* |
| **Supplementary Results** |  |
| Patient Characteristics | *page 6* |
| Accuracy of Tm Mapping | *page 6* |
| Predictive Value of Tm Mapping for POAI | *page 7* |
| **Supplementary Appendixes** |  |
| Author Contribution | *page 9* |
| **Supplementary Figures and Tables** |  |
| Supplementary Figure legend (for Fig. 1a) | *page 10* |
| Supplementary Figure 1 (Fig. S1) | *page 10* |
| Supplementary Table 1 (Table S1) | *page 11* |
| Supplementary Table 2 (Table S2) | *page 13* |
| **References** | *page 14* |

**Supplementary Methods**

**Patient Enrolment and Data Collection**

This study was approved by the Ethics Committee of the University of Toyama (approval no. R2017018) and conducted in accordance with the Declaration of Helsinki and the STROBE guidelines. Specifically, we enrolled 43 potentially eligible patients at our institution between August 2019 and May 2020. The inclusion criteria were as follows: (1) underwent elective pancreatoduodenectomy (PD) with pancreaticojejunostomy, and (2) provided written informed consent before surgery for the collection of clinical data and drainage fluid samples for subsequent Tm mapping analysis. The exclusion criteria were as follows: (1) PD with pancreaticojejunostomy was not performed (e.g., total pancreatectomy, PD with pancreaticogastrostomy) or (2) did not consent to the use of their data. Clinical data, including postoperative complication data, were extracted from medical records.

Postoperative intra-abdominal infection (POAI) was defined as either a clinically relevant postoperative pancreatic fistula (POPF, grade B or C according to the ISGPS 2016) or intra-abdominal fluid collection or an abscess of Clavien–Dindo grade II (i.e., requiring antibiotics for peritonitis) or higher and was diagnosed via physical examination or diagnostic imaging after ruling out postoperative cholangitis or other postoperative infections^1, 2^.

The surgical procedure and postoperative management were standardized at our institution, with pancreaticojejunostomy performed using the modified Blumgart method. Drainage tubes were placed at both the anterior cephalic and dorsal caudal sides of the anastomosis^3^. Cefazolin or cefmetazole was administered three days post-operatively for prophylactic purposes. Clinical decision-making regarding drain removal and therapeutic antibiotic administration was based on laboratory tests of blood and drainage fluid on postoperative day (POD) 3 and contrast-enhanced computed tomography on POD4.

**Sample Collection and Handling**

Drainage fluid was collected directly from the drainage tubes on POD1 and POD3, avoiding collection from drainage bags to minimize contamination and prevent bacterial overgrowth outside the body. All the samples were immediately frozen at −80°C until analysis.

**Melting Temperature (Tm) Mapping Method**

Tm mapping was performed as previously described^4^. Briefly, bacterial DNA was extracted from clinical samples, and nested PCR was conducted using seven pairs of partially mismatched universal primers targeting variable regions of the 16S rRNA gene^5^. PCR was first performed using a eukaryote-derived thermostable DNA polymerase (Mitsui Chemicals, Tokyo, Japan) free from bacterial DNA contamination^6^. All reactions were carried out using the Rotor-Gene Q system (Qiagen, Hilden, Germany), which enables precise temperature control in 0.5°C increments. Following the first PCR cycle, nested PCR produced seven amplicons whose Tm values were plotted and compared (i.e., “mapped”) against a reference database to identify bacterial species. The relative bacterial load was estimated according to an *Escherichia coli* standard curve (5 × 10e2 to 5 × 10e4 colony forming units (CFU)/mL).

**Statistical Analysis**

This was an observational study, and no established target sensitivity or specificity was available for the Tm mapping method. Given the low reported probability of positive Gram staining of drainage fluid (approximately 10%)^7^ and the wide variability in culture positivity rates^8, 9^, the required sample size was calculated assuming a positive culture rate of 30%, aiming for a 95% confidence interval (CI) with a total width of 0.3 (±15%). Using the continuity-corrected Wald method in R version 3.5.3, the required sample size was estimated at 43. This was corroborated using the `ssize.propCI` function in the `MKpower` package in R version 4.3.3, which returned an estimate of 42.3.

Binomial variables were compared using Pearson’s chi-square test. Continuous variables are presented as the means plus standard deviations or medians plus interquartile ranges and were analysed using Student’s t test or the Mann–Whitney U test in accordance with their distribution. Logistic regression analysis was performed to identify predictors of POAI development, with odds ratios (ORs) and 95% CIs calculated. A P value < 0.05 was considered to indicate statistical significance. All the statistical analyses and visualizations except for sample size estimation were conducted using R version 4.3.3 (The R Foundation for Statistical Computing, Vienna, Austria).

**Supplementary Results**

**Patient Characteristics**

Among the 43 enrolled patients, one patient was excluded due to undergoing total pancreatectomy. The characteristics of the 42 included patients are presented in **Table S1**. The mean age was 71 years, and there were 24 males and 18 females. Among the 27 patients who developed POAI, four patients experienced intra-abdominal abscesses that needed additional drainage with radiologic intervention. No cases of postoperative readmission to the intensive care unit with organ failure or death occurred within 90 days of the procedure. Patients with POAI were more likely to have a soft pancreatic texture, a shorter main pancreatic duct diameter, and a pancreatic tube. For perioperative antibiotic administration, cephazolin was administered to 28 patients, and cefmetazole was administered to 10 patients. Among the postoperative variables, C-reactive protein (CRP) on POD3, white blood cell count (WBC) on POD3, bacterial load by Tm mapping on POD3, drain fluid amylase (DFA) on POD1 and POD3, and ineffective coverage by administered antibiotics on POD1 appeared to be associated with the risk of developing POAI.

**Accuracy of Tm Mapping**

We tested the accuracy of the Tm mapping method using conventional culture results as the reference standard. A total of 81 drainage fluid samples were collected from the 42 patients at POD1 and POD3; three samples were missing for Tm mapping on POD3. The culture results for drainage fluid collected on POD1 were typically available on POD6 (median, range 3–10 days), and those on POD3 were also available on POD6 (median, range 6–11 days). The most frequently isolated bacteria were *Enterococcus faecium* (n = 6) and *Enterobacter cloacae* (n = 5) (**Fig. 1a**).

Receiver operating characteristic (ROC) curve analysis indicated an optimal Tm mapping bacterial load cut-off value at POD3 of 1,180 CFU/mL, yielding a sensitivity of 0.600 and a specificity of 0.867 (**Fig. S1**). Based on these results, we defined a bacterial count of more than 1,000 CFU/mL as clinically relevant for intra-abdominal infection in this study. Using this threshold, the sensitivity and specificity values of the quantitative Tm mapping test were 0.615 (0.316–0.861) and 0.759 (0.565–0.897) for POD1 and 0.826 (0.612–0.950) and 0.812 (0.544–0.960) for POD3, respectively. Note that all tests in which the bacterial load was ≤ 1,000 CFU/mL by Tm mapping were categorized as negative. With respect to qualitative tests of Tm mapping, among patients with positive culture results, the bacteria identified by Tm mapping were identical to those detected by culture in six samples at POD1 and 12 samples at POD3, corresponding to 14 patients (true positive, **Fig. 1a**). Among patients with negative culture results, bacteria were not detected by Tm mapping identically in 22 samples at POD1 and 12 samples at POD3 (true negative; **Fig. 1a**).

**Predictive Value of Tm Mapping for POAI**

We next assessed the predictive value of Tm mapping for POAI compared with DFA. DFA and bacterial loads were greater in patients who developed POAI than in those who did not (**Fig. 1b**). Notably, bacterial loads increased from POD1 to POD3 only in those who developed POAI, whereas DFA values uniformly decreased from POD1 to POD3, regardless of whether the patients developed POAI. In particular, five patients developed POAI without CR-POPF, despite DFA levels on POD3 being less than 396 U/mL, which corresponds to three times the upper limit of normal serum amylase levels. Interestingly, all five patients had bacterial loads greater than 1,000 CFU/mL on POD3, suggesting a complementary relationship between bacterial load and DFA levels with respect to POAI prediction (depicted as black dots and lines in **Fig. 1b**).

To assess the potential usefulness of Tm mapping, we developed a preliminary model for predicting POAI. Logistic regression model analysis revealed several risk factors for the development of POAI. In addition to frequently used postoperative parameters such as DFA, WBC, and CRP, the bacterial load determined by Tm mapping and the ineffective antibiotic coverage for the bacteria isolated by culture at POD1 were identified as potentially good predictors of the risk of developing POAI (**Table S2**). A generalized linear regression model (comprehensive model) was subsequently developed by incorporating DFA, WBC, CRP and bacterial load on POD3 and ineffective antibiotic coverage on POD1. ROC analysis revealed that the area under the curve (AUC) for this comprehensive model was 0.981 (95% CI, 0.951–1.000), which was superior to those of models based solely on clinical variables acquired on POD3 (DFA, WBC, and CRP) (**Fig. 1c**).

**Supplementary Appendixes**

**Author contributions**

Haruyoshi Tanaka (Conceptualization, Data curation, Formal analysis, Investigation, Project administration, Resources, Supervision, Validation, Visualization, Writing - original draft), Mina Fukasawa (Data curation, Investigation, Methodology, Resources, Validation, Visualization, Writing - original draft), Nana Kimura (Data curation, Investigation, Methodology, Resources, Writing - review & editing), Kosuke Mori (Data curation, Investigation, Resources, Writing - review & editing), Koshi Matsui (Data curation, Investigation, Methodology, Project administration, Resources, Writing - review & editing), Ayaka Itoh (Data curation, Investigation, Resources, Writing - review & editing), Katsuhisa Hirano (Data curation, Investigation, Resources, Writing - review & editing), Toru Watanabe (Data curation, Investigation, Resources, Writing - review & editing), Yoshihiro Shirai (Data curation, Investigation, Resources, Writing - review & editing), Kentaro Nagaoka (Conceptualization, Methodology, Resources, Writing - review & editing), Kazuto Shibuya (Data curation, Investigation, Resources, Writing - review & editing), Isaku Yoshioka (Data curation, Investigation, Resources, Writing - review & editing), Hideki Niimi (Conceptualization, Methodology, Project administration, Resources, Software, Supervision, Writing - review & editing), and Tsutomu Fujii (Conceptualization, Funding acquisition, Project administration, Resources, Supervision, Writing - review & editing). All authors approved the final version of the manuscript.

**Supplementary Figures and Tables**

**Supplementary Figure legend (for Fig. 1a);** Full names of bacterial genera and species: *E. faecalis, Enterococcus faecalis; C. glabrata, Candida glabrata; S. caprae, Staphylococcus caprae; E. cloacae; Enterobacter cloacae; E. faecium, Enterococcus faecium; A. baumannii; Acinetobacter baumannii; C. freundii; Citrobacter freundii; K. aerogenes; Klebsiella aerogenes; P. aeruginosa; Pseudomonas aeruginosa; C. albicans, Candida albicans;* and *S. epidermidis, Staphylococcus epidermidis.*





**Fig. S1** Receiver curve for bacterial load on POD3 measured by Tm mapping, showing the optimal cut-off value (in colony formation units/mL) for predicting postoperative intra-abdominal infection.

**Table S1.** Baseline characteristics and operative outcomes of the participants

| **Variables** | **Overall** | **non-POAI** | **POAI** | ***P*** |
| --- | --- | --- | --- | --- |
|  | N = 42 | N = 15 | N = 27 |  |
| **Preoperative variables** |  |  |  |  |
| Sex ratio (M:F) | 24:18 | 9:6 | 15:12 | 1.000 |
| Age (years), mean (SD) | 71.0 (10.1) | 76.6 (6.2) | 67.8 (10.6) | 0.006 |
| ASA–PS, n |  |  |  | 0.737 |
| I | 5 | 1 | 4 |  |
| II | 29 | 11 | 18 |  |
| III | 8 | 3 | 5 |  |
| BMI, mean (SD) | 22.4 (3.5) | 21.5 (3.3) | 22.9 (3.5) | 0.231 |
| Primary disease |  |  |  | 0.092 |
| IPMN | 13 | 6 | 7 |  |
| Pancreatic cancer | 12 | 6 | 6 |  |
| Biliary tract cancer | 11 | 1 | 10 |  |
| PanNEN | 3 | 0 | 3 |  |
| others | 3 | 2 | 1 |  |
| Biliary drainage | 18 | 5 | 13 | 0.546 |
| **Intraoperative variables** |  |  |  |  |
| Operative time (min), median [IQR] | 494 [460, 541] | 485 [449, 558] | 494 [462, 529] | 0.896 |
| Blood loss (mL), median [IQR] | 475 [253, 720] | 595 [493, 788] | 350 [200, 640] | 0.101 |
| Blood infusion | 1 | 0 | 1 | 1.000 |
| Portal vein resection | 9 | 4 | 5 | 0.922 |
| Arterial resection | 1 | 0 | 1 | 1.000 |
| Soft pancreatic texture | 31 | 8 | 23 | 0.060 |
| MPD (mm), mean (SD) | 4.1 (2.3) | 5.1 (2.8) | 3.6 (1.8) | 0.041 |
| Pancreatic tube placement | 32 | 8 | 24 | 0.027 |
| **Postoperative variables** |  |  |  |  |
| CRP (U/mL) |  |  |  |  |
| on POD1, mean (SD) | 8.3 (2.4) | 7.6 (1.8) | 8.7 (2.7) | 0.185 |
| on POD3, mean (SD) | 20.0 (7.3) | 15.9 (5.0) | 22.2 (7.5) | 0.006 |
| WBC (10e3/µL) |  |  |  |  |
| on POD1, median [IQR] | 8.91 [7.91, 10.9] | 8.5 [6.12, 9.75] | 9.25 [8.07, 11.5] | 0.096 |
| on POD3, median [IQR] | 9.70 [8.24, 12.25] | 7.72 [6.94, 9.33] | 11.01 [9.49, 13.06] | 0.001 |
| Bacterial load (CFU/mL) |  |  |  |  |
| on POD1, median [IQR] | 175 [28, 2563] | 75 [25, 200] | 306 [59, 4605] | 0.083 |
| on POD3, median [IQR] | 425 [25, 4655] | 50 [25, 505] | 2400 [25, 80750] | 0.028 |
| Drain fluid amylase (U/mL) |  |  |  |  |
| on POD1, median [IQR] | 2046 [550, 6235] | 504 [86, 1202] | 4193 [1681, 7110] | <0.001 |
| on POD3, median [IQR] | 655 [187, 1803] | 155 [66, 544] | 1220 [593, 2329] | <0.001 |
| Ineffective coverage by antibiotics |  |  |  |  |
| on POD1 | 12 | 1 | 11 | 0.047 |
| on POD3 | 21 | 7 | 14 | 1.000 |
| **Postoperative outcomes** |  |  |  |  |
| Postoperative complication Clavien–Dindo classification | |  |  | <0.001 |
| None | 10 | 10 | 0 |  |
| I | 4 | 4 | 0 |  |
| II | 15 | 1 | 14 |  |
| IIIa | 12 | 0 | 12 |  |
| IIIb | 1 * | 0 | 1 |  |
| Postoperative death † | 0 | 0 | 0 | 1.000 |
| Values are n (%) unless otherwise indicated. POAI, postoperative intra-abdominal infection; ASA–PS, American Society of Anesthesiologists–Performance Status; BMI, body mass index; IPMN, intraductal papillary mucinous neoplasm; PanNEN, Pancreatic neuroendocrine neoplasm; MPD, main pancreatic duct (diameter); CRP, C-reactive protein; WBC, white blood cell count; POD, postoperative day; CFU, colony-forming unit; IQR, interquartile range; SD, standard deviation; * reoperation for obstructive ileus; † within 90 days post-operative. | | | | |

**Table S2.** Logistic regression model analysis of risk factors for the development of POAI

| **Variables** | **Odds ratio** | **95% CI** | ***P*** |
| --- | --- | --- | --- |
| Age (>80 years old) | 0.34 | 0.059, 1.810 | 0.207 |
| Sex (male) | 0.83 | 0.223, 2.987 | 0.780 |
| Texture of pancreas (soft) | 5.03 | 1.210, 23.9 | 0.031 |
| Preoperative biliary drainage (yes) | 1.86 | 0.513, 7.34 | 0.355 |
| Primary disease (Pancreatic cancer) | 0.43 | 0.105, 1.70 | 0.227 |
| Operative time (>10 hours) | 0.70 | 0.132, 4.01 | 0.667 |
| Intraoperative blood loss (>1000 mL) | 0.81 | 0.120, 6.77 | 0.831 |
| Intraoperative blood transfusion (yes) | 2.44 | 0.319, 50.2 | 0.446 |
| Vessel resection (yes) | 0.87 | 0.201, 4.03 | 0.850 |
| WBC count at POD3 (>10×10e3/µL) | 13.0 | 2.83, 95.6 | 0.003 |
| CRP on POD3 (>20 mg/dL) | 6.80 | 1.69, 35.5 | 0.012 |
| DFA on POD1 (>2000 U/mL) | 6.60 | 1.68, 29.6 | 0.009 |
| DFA on POD3 (>396 U/mL) | 15.4 | 3.32, 114.8 | 0.002 |
| Ineffective antibiotic coverage on POD1 | 9.63 | 1.57, 187.3 | 0.041 |
| Ineffective antibiotic coverage on POD3 | 1.23 | 0.346, 4.46 | 0.748 |
| Bacterial load by Tm mapping on POD1 (>250 CFU/mL) | 5.82 | 1.45, 30.2 | 0.020 |
| Bacterial load by Tm mapping on POD3 (>1000 CFU/mL) | 9.75 | 2.11, 71.6 | 0.008 |
| POAI, postoperative intra-abdominal infection; POD, postoperative day; CI, confidence interval; WBC, white blood cell; CRP, C-reactive protein; DFA, drain fluid amylase; CFU, colony formation unit. | | | |

**References**

1. Bassi C, Marchegiani G, Dervenis C, Sarr M, Abu Hilal M, Adham M, et al. The 2016 update of the International Study Group (ISGPS) definition and grading of postoperative pancreatic fistula: 11 Years After. Surgery. 2017;161(3):584-91.

2. Dindo D, Demartines N, Clavien PA. Classification of surgical complications: a new proposal with evaluation in a cohort of 6336 patients and results of a survey. Ann Surg. 2004;240(2):205-13.

3. Fujii T, Sugimoto H, Yamada S, Kanda M, Suenaga M, Takami H, et al. Modified Blumgart anastomosis for pancreaticojejunostomy: technical improvement in matched historical control study. J Gastrointest Surg. 2014;18(6):1108-15.

4. Niimi H, Ueno T, Hayashi S, Abe A, Tsurue T, Mori M, et al. Melting Temperature Mapping Method: A Novel Method for Rapid Identification of Unknown Pathogenic Microorganisms within Three Hours of Sample Collection. Sci Rep. 2015;5:12543.

5. Wilson KH, Blitchington RB, Greene RC. Amplification of bacterial 16S ribosomal DNA with polymerase chain reaction. J Clin Microbiol. 1990;28(9):1942-6.

6. Niimi H, Mori M, Tabata H, Minami H, Ueno T, Hayashi S, et al. A novel eukaryote-made thermostable DNA polymerase which is free from bacterial DNA contamination. J Clin Microbiol. 2011;49(9):3316-20.

7. Chinnock B, Fox C, Hendey GW. Gram's stain of peritoneal fluid is rarely helpful in the evaluation of the ascites patient. Ann Emerg Med. 2009;54(1):78-82.

8. Yang F, Jin C, Li J, Di Y, Zhang J, Fu D. Clinical significance of drain fluid culture after pancreaticoduodenectomy. J Hepatobiliary Pancreat Sci. 2018;25(11):508-17.

9. Yamashita K, Kato D, Sasaki T, Shiwaku H, Ishii F, Naito S, et al. Contaminated drainage fluid and pancreatic fistula after pancreatoduodenectomy: A retrospective study. Int J Surg. 2018;52:314-9.
